# Cobalt-Catalyzed C–Se Bond Activation: Cross-Coupling of Organoselenides with Grignard Reagents

**DOI:** 10.3390/molecules30214232

**Published:** 2025-10-30

**Authors:** Tais V. de Souza Pinto, Kamilly D. V. P. Sobrinho, Maria E. C. da Silva, Sandynara A. de Oliveira, Andreia P. V. de Jesus, Tereza A. N. Ribeiro, Leonardo G. de Vasconcelos, Paulo T. de Sousa Júnior, Sumbal Saba, Jamal Rafique, André L. Stein

**Affiliations:** 1 Laboratório de Pesquisa de Produtos Naturais, Instituto de Química, Universidade Federal de Mato Grosso—UFMT, Cuiabá 78060-900, MT, Brazil; 2 Instituto de Química (INQUI), Universidade Federal do Mato Grosso do Sul—UFMS, Campo Grande 79074-460, MS, Brazil; 3 Laboratório de Síntese Sustentável e Organocalcogênio (LABSO), Instituto de Química (IQ), Universidade Federal de Goiás—UFG, Goiânia 74690-900, GO, Brazil; sumbalsaba@ufg.br

**Keywords:** selenium, cross-coupling, Grignard reaction, cobalt catalysis

## Abstract

Herein, we report a cobalt-catalyzed cross-coupling of organoselenides with Grignard reagents using simple CoCl_2_ as the catalyst. This efficient method accommodates a broad scope of selenides, including vinyl, aryl, heteroaryl, and alkynyl derivatives, under mild and ligand-free conditions. Remarkably, the reaction proceeds efficiently without additives and afforded the desired products in moderate to good yields within just 3 h.

## 1. Introduction

Cross-coupling reactions represent one of the most powerful tools for constructing complex molecules, particularly for forming Csp^2^–Csp^2^ bonds [1]. The wide-ranging application of these reactions in the synthesis of bioactive molecules—particularly in the pharmaceutical and agrochemical industries—was recognized by the 2010 Nobel Prize in Chemistry [2,3]. These transformations typically involve a metal-catalyzed reaction between a nucleophilic organometallic reagent and an electrophilic substrate bearing a halide or pseudohalide leaving group. Prominent examples include the Suzuki–Miyaura [4], Kumada–Tamao–Corriu [5], Sonogashira [6], Stille [7], Negishi [8], and Hiyama [9] reactions.

In the past few years, significant efforts have been devoted to developing sustainable protocols that involve use of various solvents, catalysts, ligands, and additives to accommodate more readily available substrates (C–X) and less reactive organometallic reagents (e.g., Sn, Al, Zn, B, Si) [10]. However, Grignard reagents remain widely used due to their high reactivity and ease of preparation [5]. The application of Grignard reagents has evolved far beyond their classical role in nucleophilic addition, emerging as versatile bases for direct C–H metallation. Notably, their utility in this field is often enhanced by their surprisingly mild and selective basicity compared to stronger alternatives like alkyllithiums, which can lead to over-metallation or substrate decomposition. This strategic use of Grignard reagents as moderate bases has been powerfully demonstrated in recent years for the catalytic functionalization of a range of challenging substrates [11,12,13]. As a result, numerous cross-coupling protocols involving Grignard reagents and various substrates, catalyzed by nickel, palladium, or cobalt, have been reported in recent years [14,15,16].

Organoselenium compounds have attracted considerable attention, primarily due to their diverse biological activities [17,18,19]. These compounds can be selectively introduced into organic substrates through both nucleophilic and electrophilic pathways, often with high chemo-, regio-, and stereo-control [20,21]. Selenium compounds are generally stable in air and in common organic solvents, further enhancing their synthetic utility. Owing to the ability of selenium to exhibit oxidation states ranging from −2 to +6, these compounds display a wide range of structural diversity. Representative classes include diselenides, selenides, selenoxides, selenones, vinyl selenides, and selenoacetylenes [22].

A significant focus in chalcogen chemistry involves heterocyclic systems, where functionalization strategies range from simple reactions with elemental selenium or diselenides to sophisticated transition-metal-catalyzed transformations [23,24,25]. Among the most powerful methods, transition-metal-catalyzed cyclization of simple acyclic precursors stands out as a particularly attractive approach for the direct construction of chalcogen-containing heterocycles [26,27]. Although previous studies have demonstrated promising results, some of them have space of improvement considering the use of precious metals, costly catalysts, ligand, and high reagent loadings. To address these shortcomings, the development of a more sustainable and efficient alternative is urgently needed.

**Scheme 1:**
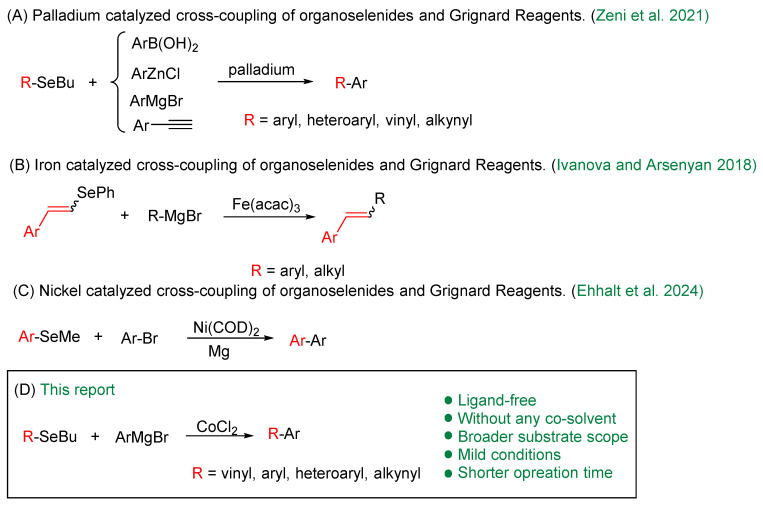
Metal-catalyzed cross-coupling of organoselenides and Grignard Reagents [26,27,28].

Although organohalides (C–X, where X = I, Br, Cl) and pseudohalides (X = OC(O)R, OTs, OTf, NC(O)R) are the most electrophiles in cross-coupling reactions [28,29]—mainly due to their low bond dissociation energies—organoselenium compounds have emerged as promising alternatives (Scheme 1). This potential is exemplified by the work reported by Zeni and co-authors in 2015, which describes the use of various classes of organoselenium compounds as substrates in palladium-catalyzed cross-coupling reactions, including Negishi, Suzuki, Kumada, and Sonogashira protocols [30]. Further advances include Silveira and co-workers’ iron-catalyzed (Fe(acac)_3_) cross-coupling of vinylic selenides (both *E* and *Z* isomers) and tellurides with Grignard reagents, which proceeds with complete retention of configuration and excellent yields [31]. Most recently, a nickel-catalyzed cross-coupling reaction was developed using an aryl alkyl selenide and an aryl bromide in the presence of 3 equivalents of magnesium [32]. This one-pot transformation proceeds via cleavage of the C–Se bond, affording the desired biaryls in moderate to good yields, while avoiding the pre-preparation of Grignard reagents.

As part of our ongoing interest in organoselenium chemistry and in the development of new synthetic methodologies [33,34,35,36,37,38,39,40], we herein report a cobalt-catalyzed cross-coupling of diverse organoselenides (vinyl, heteroaryl, aryl, and alkynyl) with Grignard reagents using CoCl_2_ as a simple, efficient catalyst.

## 2. Results & Discussion

For the optimization, (*Z*)-butyl(styryl)selenide **1a** and freshly prepared (4-methoxyphenyl) magnesium bromide **2a** (1 M solution) were selected as model substrates (Table 1). Initially, **2a** (2 equiv.) were added dropwise to a solution of **1a** (0.25 mmol) in THF (1 mL) at 0 °C. After 2 h, product **3a** was isolated in 50% yield (entry 1). We then evaluated common co-solvents for Grignard cross-coupling reactions, but THF/NMP (2:1) and THF/Et_3_N (2:1) mixtures did not improve the yield (entries 2–3). Similarly, conducted reaction at room temperature proved ineffective (entries 4–5), with incomplete consumption of **1a** observed in all cases.

Notably, using 5 mol% of catalyst at 0 °C led to a significant increase in the yield of product **3a**, whereas 2 mol% proved insufficient (entries 6–7). As expected, in the absence of the cobalt catalyst, only the starting material was recovered (Table 1, entry 8). Further optimization revealed that extending the reaction time to 3 h provided **3a** in 73 and 83% yield for 2 mol% and 5 mol % catalytic loading, respectively (entry 9–10). Prolonging the reaction beyond 3 h did not further improve the yield. Temperature control was critical, as performing the reaction at room temperature substantially reduced the yield (entry 11). The use of an excess of Grignard reagent (2 equiv.) was necessary due to the competitive formation of a biaryl byproduct alongside product **3a**. Although 1.5 equivalents of **2a** appeared to be sufficient, we opted to maintain the use of 2 equivalents for consistency.

With the optimized conditions in hand (Table 1, entry 10), we next explored the scope and limitations of the method by applying it to a range of substrates (Table 2). First, we examined various aryl Grignard reagents (freshly prepared) with vinyl selenide **1a**. Aryl Grignard reagents bearing electron-donating groups (**2a** and **2b**) reacted efficiently, providing higher yields compared to phenylmagnesium bromide (**2c**) (Table 2, entries 1–3). In contrast, the presence of an electron-withdrawing substituent, such as fluorine, diminished reactivity, affording product **3b** in only 63% yield (Table 2, entry 4). Notably, alkyl Grignard reagents generated multiple byproducts with similar polarity, preventing isolation of the desired products. Similarly, when substrate **1b** was employed, product **3e** and the corresponding biaryl byproduct co-formed an inseparable mixture, making purification and yield estimation challenging (Table 2, entry 5). However, when Grignard reagent **2b** was used, product **3f** was isolated in 87% yield (Table 2, entry 6).

Other vinyl selenides, such as **1c**, **1d**, and **1e**, also reacted satisfactorily with reagent **2b** (Table 2, entries 7–9). Interestingly, the reaction also proved efficient for the coupling between a divinylic selenide and Grignard reagents. In this case, the use of 4 equivalents of Grignard reagent **2a** led to the formation of product **3a** in 73% yield (Table 2, entry 10). The protocol tolerated functional groups such as alcohols, substrate **1g** reacted successfully with **2a**, albeit requiring 3 equivalents of the Grignard reagent (Table 2, entry 11).

**Scheme 2:**
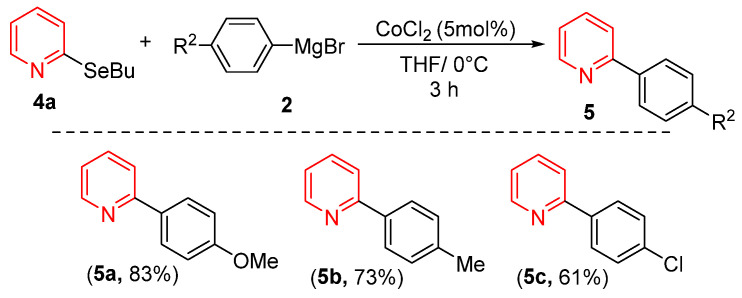
Cross-Coupling of 2-(buthylselanyl)pyridine with Grignard Reagents.

Considering that heterocycle-based organoselenium compounds represent one of the most important classes of selenium-containing molecules, we investigated the reactivity of pyridine derivative **4a** with various Grignard reagents under our optimized conditions. As shown in Scheme 2, both electron-rich and electron-deficient Grignard reagents successfully coupled with **4a**, yielding products **5a**–**5c** in good yields. Notably, Grignard reagents bearing electron-donating groups consistently provided superior yields compared to their electron-withdrawing counterparts.

**Scheme 3:**
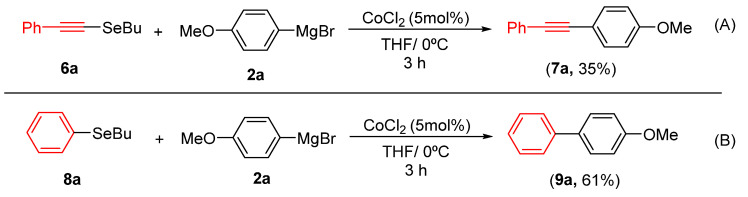
Reactivity of Grignard Reagent **2a**. (**A**) with **6a**; (**B**) with **8a**.

To further explore the synthetic utility of and demonstrate the potential of alkynyl and aryl selenide derivatives as starting materials, we evaluated their reactivity in cobalt-catalyzed cross-coupling reactions. Under standard conditions, compound **6a** coupled with Grignard reagent **2a** to afford product **7a** in 35% yield (Scheme 3A). More encouragingly, the reaction of aryl selenide **8a** furnished the corresponding biaryl **9a** in 61% yield (Scheme 3B), demonstrating the particular effectiveness of this substrate class.

**Scheme 4:**
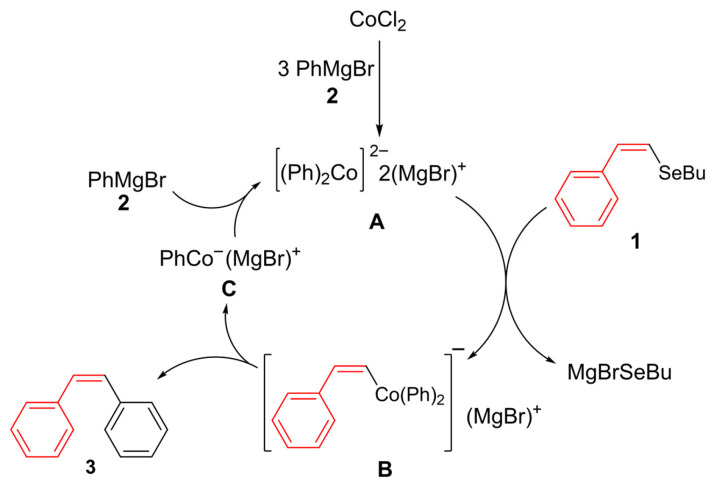
A possible mechanism.

Building upon the foundational work by Oshima and co-workers [41,42] on cobalt-catalyzed cross-coupling reactions involving Grignard reagents, and later advancements by Hayashi [43], we were encouraged to propose a detailed mechanism (Scheme 4). In these studies, a complex formed in situ from cobalt salts and at least 4 equivalents of PhMgBr drastically altered the reaction outcome, with the coupling product being preferentially obtained over biphenyl. In our proposal, CoCl_2_ is first reduced to a cobalt(0) complex by 4 equivalents of aryl Grignard reagent **2.** This complex then undergoes oxidative addition to form a bis-aryl cobalt(III) intermediate **B**, which subsequently undergoes reductive elimination to give the product **3** and monoaryl-ate complex **C**. Finally, the reaction of **C** with **2** regenerates di-ate complex **A**.

## 3. Materials and Methods

The reagents used were purchased from Aldrich Chemical Co. (Cajamar, Brazil) and used without purification. All the solvents used were of PA grade and used without purification. Analytical thin-layer chromatography analysis was carried out using Merck DC Kieselgel 0 F254 (Merck, Darmstadt, Germany) (230–400 mesh) aluminum plates coated with treated silica. For visualization, TLC plates were either placed under ultraviolet light, or stained with iodine vapor, or acidic vanillin. Most reactions were monitored by TLC for disappearance of starting material. Chromatographic columns (LabGlass, Sao Paulo, Brazil) were filled with silica gel 60 (230–400 mesh ASTM). Nuclear magnetic resonance (NMR) spectra were recorded using a Bruker Ascend 500TM spectrometer or Bruker DPX 300 NMR spectrometer (Bruker, Rheinstetten, Germany), with the chemical shifts (*δ*) set in parts per million (ppm), referenced by the residual solvent peak. The multiplicity of each peak is designated by the following abbreviations: s (singlet), d (doublet), dd (doublet of doublet), t (triplet), quint (quintet), sext (sextet) and m (multiplet). The coupling constants (*J*) are reported in Hertz (Hz).

### General Procedure for the Cobalt-Catalyzed Cross-Coupling Reaction of Organoselenides with Organomagnesium Reagents

A mixture of the organoselenide (0.20 mmol) and CoCl_2_ (0.05 equiv.) was dissolved in THF (1 mL). Subsequently, freshly prepared organomagnesium reagent (2 equiv., 1 M solution) was then added. The mixture was stirred for 3 h at 0 °C. Afterward, the reaction mixture was allowed to warm to room temperature, diluted with ethyl acetate (3 mL), and washed with a saturated solution of NH_4_Cl (20 mL). The organic layer was separated, dried over MgSO_4_, and concentrated under reduced pressure. The crude product was purified by flash chromatography, eluting with hexane. Spectra of all the synthesized compounds are available in the Supplementary Materials (Figures S1–S28).

#### Spectral Data of Synthesized Products (**3a**–**3j**, **5a**–**5c**, **7a**, **9a**)

(*Z*)*-1-methoxy-4-styrylbenzene* **3a** [44]. Yield: 83% (0.035 g), colorless liquid. RMN ^1^H (CDCl_3_, 500 MHz): δ = 7.27–7.17 (m, 7H); 6.75 (d, *J* = 8.7 Hz, 2H); 6.54–6.48 (m, 2H); 3.77 (s, 3H). RMN ^13^C (CDCl_3_, 125 MHz): δ = 158.60; 135.56; 137.57; 130.16; 129.78; 129.67; 128.83; 128.77; 128.25; 126.9; 55.20.

(*Z*)*-1-methil-4-styrylbenzene* **3b** [45]. Yield: 87% (0.034 g), colorless liquid. RMN ^1^H (CDCl_3_, 500 MHz): δ = 7.46 (d, *J* = 7.9 Hz, 3H); 7.25–7.12 (m, 5H); 6.54 (s, 1H); 2.37 (s, 3H). RMN ^13^C (CDCl_3_, 125 MHz): δ = 138.49; 136.91; 130.41; 129.64; 129.11; 129.05; 128.99; 128.37; 128.39; 127.02; 21.25.

(*Z*)*-1,2-diphenylethene* **3c** [44]. Yield: 72% (0.026 g), colorless liquid. RMN ^1^H (CDCl_3_, 500 MHz): δ = 7.65 (d, *J* = 7.2 Hz, 1H); 7.50 (t, *J* = 7,7 Hz, 1H) 7.43–7.39 (m, 1H); 7.31–7.23 (m, 7H); 6.66 (s, 2H). RMN ^13^C (CDCl_3_, 125 MHz): δ = 137.29, 130.29, 128.92, 128.25, 127.13.

(*Z*)*-1-fluoro-4-styrylbenzene* **3d** [44]. Yield: 63% (0.024 g), colorless liquid. RMN ^1^H (CDCl_3_, 500 MHz): δ = 7.49–7.46 (m, 1H); 7.22–7.17 (m, 5H); 7.12–7,09 (m, 1H); 6,91–6.87 (m, 2H); 6,56 (q, *J* = 12.1 Hz, 2H). RMN ^13^C (CDCl_3_, 125 MHz): δ = 161,97 (d, *J* = 246.6 Hz); 137.24; 1336.37 (d, *J* = 3.4 HZ); 130.73 (d, 7.9 Hz); 130.46; 129.28, 129.03; 128.51; 127.40; 115.45 (d, *J* = 21.4 Hz).

(*Z*)*-1-methoxy-4-(4-methylstyryl)benzene* **3f** [45]. Yield: 87% (0.0386 g), colorless liquid. RMN ^1^H (CDCl_3_, 500 MHz): δ = 7.21–7.14 (m, 4H); 7.03 (d, *J* = 8.0 Hz, 2H); 6.75 (d, *J* = 8.7 Hz, 2H); 6.47 (s, 2H); 2.31 (s, 3H); 3.79 (s, 3H); 2.33 (s, 3H). RMN ^13^C (CDCl_3_, 125 MHz): δ = 158.60; 136.65; 134.63; 130.28; 130.12; 129.12; 128.94; 128.76; 128.76; 113.58; 55.20; 21.25.

(*Z*)*-1-methoxy-4-(4-methylstyryl)benzene* **3g** [44]. Yield: 69% (0.031 g), colorless liquid. RMN ^1^H (CDCl_3_, 500 MHz): δ = 7.22–7.17 (m, 4H); 7.05 (d, *J* = 8.0 Hz, 2H); 6.77 (d, *J* = 8.7 Hz, 2H); 6.49 (s, 2H); 2.31 (s, 3H); 3.79 (s, 3H); 2.33 (s, 3H). RMN ^13^C (CDCl_3_, 125 MHz): δ = 158.68; 136.81; 134.80; 130.29; 130.12; 129.28; 129.11; 128.93; 128.90; 113.75; 55.35; 21.42.

(*Z*)*-1-choro-4-(4-methoxystyryl)benzene* **3h** [46]. Yield: 77% (0.038 g), colorless liquid. RMN ^1^H (CDCl_3_, 500 MHz): δ = 7.17 (d, *J* = 7.3 Hz, 4H); 7.15 (d, *J* = 8.6 Hz, 2H); 6.75 (d, *J* = 8.6 Hz, 2H); 6.49 (s, 2H); 6.53 (d, *J* = 12.1 Hz, 1H); 6.42 (d, *J* = 12.1 Hz, 1H); 3.79 (s, 3H). RMN ^13^C (CDCl_3_, 125 MHz): δ = 158.68; 136.81; 134.80; 130.29; 130.12; 129.28; 129.11; 128.93; 128.90; 113.75; 55.35.

(*Z*)*-1-methoxy-4-(4-(trifluoromethyl)ystyryl)benzene* **3i** [47]. Yield: 69% (0.038 g), colorless liquid. RMN ^1^H (CDCl_3_, 500 MHz): 7.46 (d, *J* = 8.1 Hz, 2H); 7.35 (d, *J* = 8.1 Hz, 2H); 7.14 (d, *J* = 8.6 Hz, 2H); 6.76 (d, *J* = 8.6 Hz, 2H); 6.63 (d, *J* = 12.2 Hz, 1H); 6.48 (d, *J* = 12.2 Hz, 1H); 3.78 (s, 3H). RMN ^13^C (CDCl_3_, 125 MHz): δ = 159.25; 141.53; 132.02; 130.38; 129.28; 129.11; 127.41; 125.37 (q, *J* = 3.8 Hz); 123.34; 113.99; 54.42.

(*Z*)*-3-(4-mehoxyphenyl)prop-2-en-1-ol* **3j** [44]. Yield: 43% (0.013 g), colorless liquid. RMN ^1^H (CDCl_3_, 500 MHz): 7.14 (d, *J* = 8.6 Hz, 2H); 6.85 (d, *J* = 8.6 Hz, 2H); 6.49 (d, *J* = 11.6 Hz, 1H); 5.78–5.73 (m, 1H); 4.41 (d, *J* = 6.4 Hz, 1H); 3.80 (s, 3H). RMN ^13^C (CDCl_3_, 125 MHz): δ = 158.75; 130.67; 130.11; 129.41; 127.15; 113.71; 59.78; 55.29.

*2-(4-methoxyphenyl)pyridine***5a** [30]. Yield: 83% (0.031 g), colorless solid. RMN ^1^H (CDCl_3_, 500 MHz): δ = 8.63 (ddd, *J* = 4.8, 1.6, 1.0 Hz, 1H); 7.95–7.91 (m, 2H); 7.71–7.63 (m, 2H); 7.15 (ddd, *J* = 7.2, 4.8, 1.3 Hz, 1H); 7.71–7.63 (m, 2H); 3.84 (s, 3H). RMN ^13^C (CDCl_3_, 125 MHz): δ = 160.65; 157.29; 149.93; 136.85; 132.24; 128.19; 121.67; 120.00; 114.41; 55.44.

*2-(p-tolyl)pyridine***5b** [48]. Yield: 73% (0.025 g), colorless solid. RMN ^1^H (CDCl_3_, 500 MHz): δ = 8.66 (ddd, *J* = 4.8, 1.6, 1.0 Hz, 1H); 7.87 (d, *J* = 8.2 Hz, 1H); 7.73–7.67 (m, 2H); 7.26 (d, *J* = 8.2 Hz, 1H); 7.18 (ddd, *J* = 6.7, 4.8, 1.6, Hz, 1H); 3.84 (s, 3H). RMN ^13^C (CDCl_3_, 125 MHz): δ = 157.70; 149.68; 139.19; 136.91; 129.69; 126.88; 122.01; 120.37; 115.36; 21.47.

*2-(4-chorophenyl)pyridine***5c** [48]. Yield: 61% (0.023 g), colorless solid. RMN ^1^H (CDCl_3_, 500 MHz): δ = 8.71 (ddd, *J* = 4.8, 1.6, 1.0 Hz, 1H); 7.96 (d, *J* = 7.9 Hz, 2H); 7.77 (td, *J* = 7.7, 1.7 Hz, 2H); 7.72 (d, *J* = 8.0 Hz, 2H); 7.47 (d, *J* = 7.9 Hz, 2H); 7.28–7.25 (m, 1H). RMN ^13^C (CDCl_3_, 125 MHz): δ = 156.42; 150.00; 138.01; 137.13; 129.16; 128.37; 122.56; 120.40.

*1-methoxy-4-(phenylethynyl)benzene***7a** [30]. Yield: 35% (0.015 g), colorless solid. RMN ^1^H (CDCl_3_, 500 MHz): δ = 7.51–7.49 (m, 2H); 7.45 (d, *J* = 8.6 Hz, 2H); 7.34–7.29 (m, 3H); 6.86. (d, *J* = 8.6 Hz, 2H); 3.81 (s, 3H). RMN ^13^C (CDCl_3_, 125 MHz): δ = 159.23; 133.20; 131.66; 128.61; 128.09; 123.66; 115.49; 114.13; 89.57; 88.23; 55.51.

*4-methoxy-1,1′-biphenyl***9a** [30]. Yield: 61% (0.022 g), white solid. RMN ^1^H (CDCl_3_, 500 MHz): δ = 7.55–7.51 (m, 4H); 7.42–7.39 (m, 2H); 7.31–7.28 (m, 1H); 6.97 (d, *J* = 8.6 Hz, 2H); 3.84 (s, 3H). RMN ^13^C (CDCl_3_, 125 MHz): δ = 159.34; 141.03; 135.44; 133.92; 128.92; 128.36; 126.94; 114.29; 55.55.

## 4. Conclusions

In summary, we have developed a practical and efficient cobalt-catalyzed cross-coupling of organoselenides with Grignard reagents under mild conditions. Under the optimized conditions, the reaction operates without any ligands or co-solvents, and good yields were achieved in just 3 h using only 5 mol% of the cobalt catalyst. This method significantly expands the scope of metal-catalyzed cross-coupling reactions involving freshly prepared Grignard reagent, accommodating not only vinyl selenides but also aryl, heteroaryl, and alkynyl derivatives. Given the facile preparation of organoselenium compounds and their inherent chemo-, regio-, and stereoselective reactivity, this transformation offers a versatile and powerful strategy for C–C bond formation.

## Figures and Tables

**Table 1:** Optimization of the cross-coupling conditions ^[a]^.

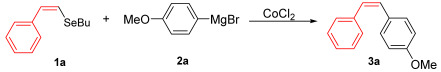				
Entry	CoCl_2_ (mol%)	Solvent	Temp. (°C)	Yield 3a (%) ^[b]^
1	10	THF	0	50
2	10	THF/NMP (2:1)	0	28
3	10	THF/Et_3_N (2:1)	0	40
4	10	THF	25	44
5	5	THF	25	46
6	5	THF	0	65
7	2	THF	0	62
8	-	THF	0	0
9	2	THF	0	73 ^[c]^
10	5	THF	0	83 ^[c]^
11	5	THF	25	58 ^[c]^
12	5	THF	0	81 ^[c,d]^

^[a]^ Reaction conditions: 0.25 mmol **1a**, 0.50 mmol (2 equiv.) freshly prepared Grignard reagent **2a**, 5 mol% CoCl_2_, in 1 mL THF, 2 h. ^[b]^ Yield of isolated product. ^[c]^ 3 h. ^[d]^ 1.5 equiv. of **2a**.

**Table 2:** Scope of the Reaction Between Vinyl Selenides and Grignard Reagents ^[a]^.

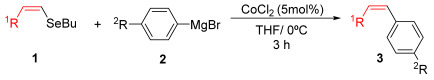			
Entry	^1^RSeBu 1	R^2^MgBr 2	Yield 3 (%) ^[b]^
1	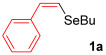	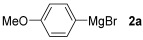	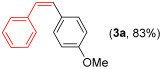
2	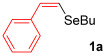	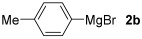	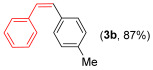
3	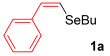	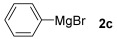	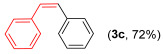
4	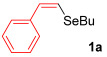	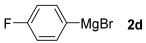	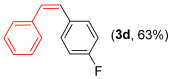
5 ^[c]^	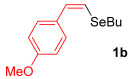	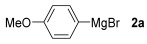	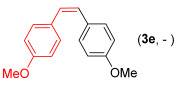
6	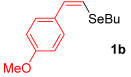	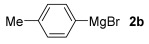	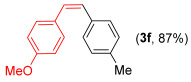
7	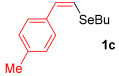	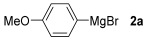	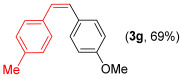
8	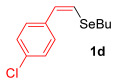	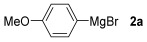	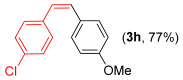
9	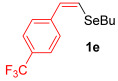	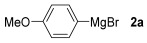	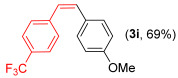
10 ^[d]^	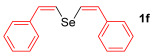	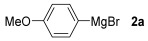	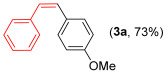
11	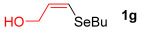	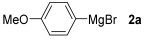	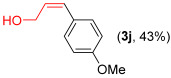

^[a]^ Reaction conditions: 0.25 mmol **1**, 0.50 mmol (2 equiv.) freshly prepared Grignard reagent **2**, 5 mol% CoCl_2_ in 1 mLTHF, 0 °C, 3 h. ^[b]^ Yield of isolated product. ^[c]^ Inseparable mixture of the product with the formed biaryl, derived from the Grignard reagent. ^[d]^ 4 equivalents of the Grignard reagent were used.

## Data Availability

The original contributions presented in this study are included in the article and Supplementary Materials. Further inquiries can be directed to the cor-responding authors.

## Supplementary Materials

The following supporting information can be downloaded at: https://www.mdpi.com/article/10.3390/molecules30214232/s1, NMR Spectra (Figures S1–S28).
