# Supplementary material for: Cobalt-Catalyzed C–Se Bond Activation: Cross-Coupling of Organoselenides with Grignard Reagents
Source: Molecules. 2025 Oct 30;30(21):4232. doi: 10.3390/molecules30214232 (PMC12610506; doi:10.3390/molecules30214232)
Supplement: Supplementary file 1 [file molecules-30-04232-s001.zip › molecules-3846197-supplementary.pdf]

Electronic Supplementary Information

Cobalt-Catalyzed C–Se Bond Activation: Cross-Coupling of Organoselenides with Grignard Reagents

Tais V. de Souza Pinto [1], Kamilly D. V. P. Sobrinho [1], Maria E. C. da Silva [2], Sandynara A. de Oliveira [1], Andreia P. V. de Jesus [1], Tereza A. N. Ribeiro [1], Leonardo G. de Vasconcelos [1], Paulo T. de Sousa Júnior [1], Sumbal Saba [3], Jamal Rafique [2,3,*] and André L. Stein [1,*]

[1] Laboratório de Pesquisa de Produtos Naturais, Instituto de Química, Universidade Federal de Mato Grosso—UFMT, Cuiabá 78060-900, MT, Brazil; taaisvitoria98@gmail.com (T.V.d.S.P.); kdvpsobrinho@gmail.com (K.D.V.P.S.); sandyaguiar@outlook.com (S.A.d.O.); andreiaapveiga@gmail.com (A.P.V.d.J.); tereza.ribeiro@ufmt.br (T.A.N.R.); leonardo.vasconcelos@ufmt.br (L.G.d.V.); pauloteixeiradesousa@gmail.com (P.T.d.S.J.)

[2] Instituto de Química (INQUI), Universidade Federal do Mato Grosso do Sul—UFMS, Campo Grande 79074-460, MS, Brazil; mariaeduarda.cardoso@hotmail.com

[3] Laboratório de Síntese Sustentável e Organocalcogênio (LABSO), Instituto de Química (IQ), Universidade Federal de Goiás—UFG, Goiânia 74690-900, GO, Brazil; sumbalsaba@ufg.br

* Correspondence: jamal.rafique@ufms.br or jamal.chm@gmail.com (J.R.); andre.stein@ufmt.br (A.L.S.)

Contents

Copies of (^{1}H and ^{13}C) NMR spectra...S2

SELECTED SPECTRA

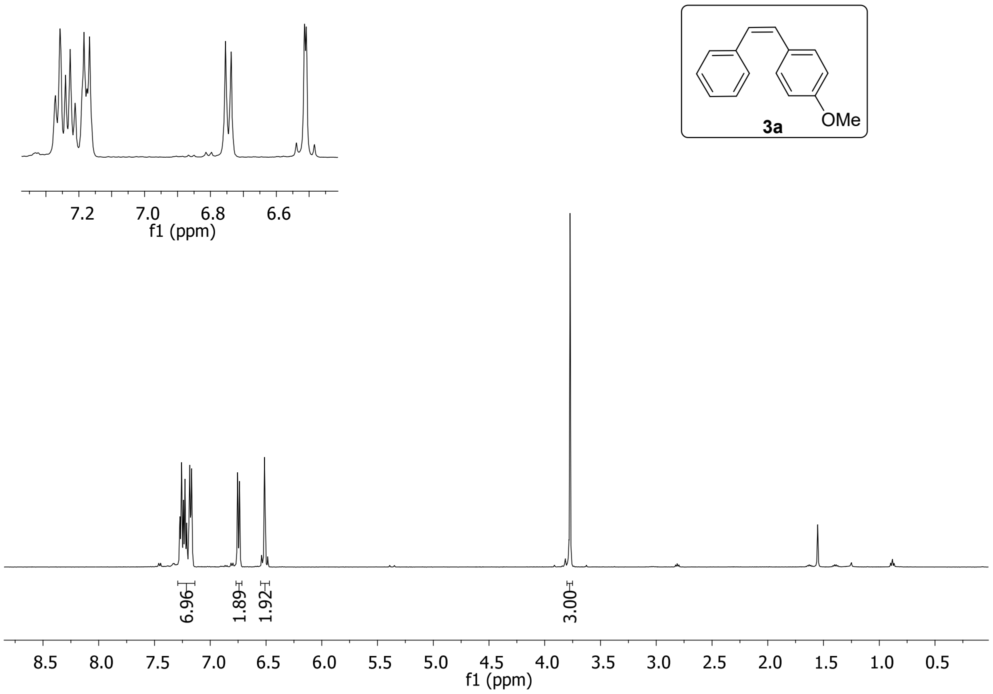

Figure S1. ^{1}H-NMR Spectra of the compound **3a,** in $CDCl_3$, 500 MHz

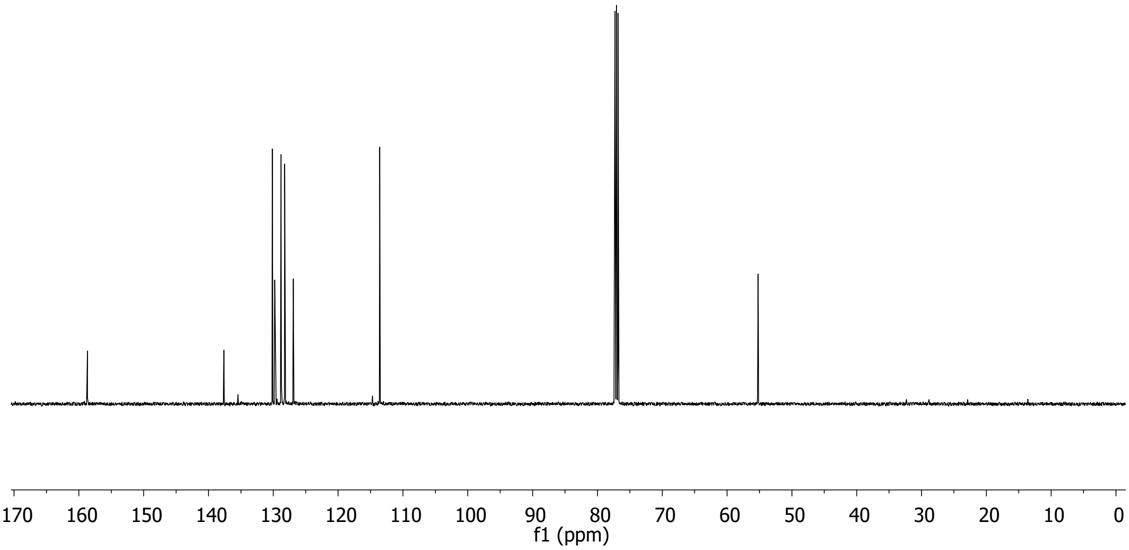

Figure S2. ^{13}C-NMR Spectra of the compound **3a**, in $CDCl_3$, 125 MHz

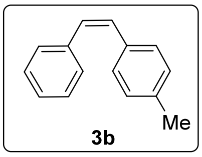

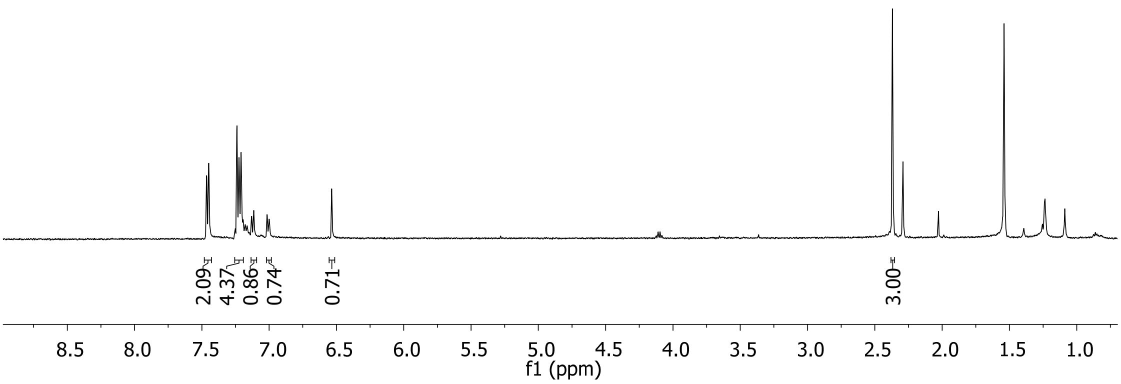

Figure S3. ^{1}H-NMR Spectra of the compound **3b,** in $CDCl_3$, 500 MHz

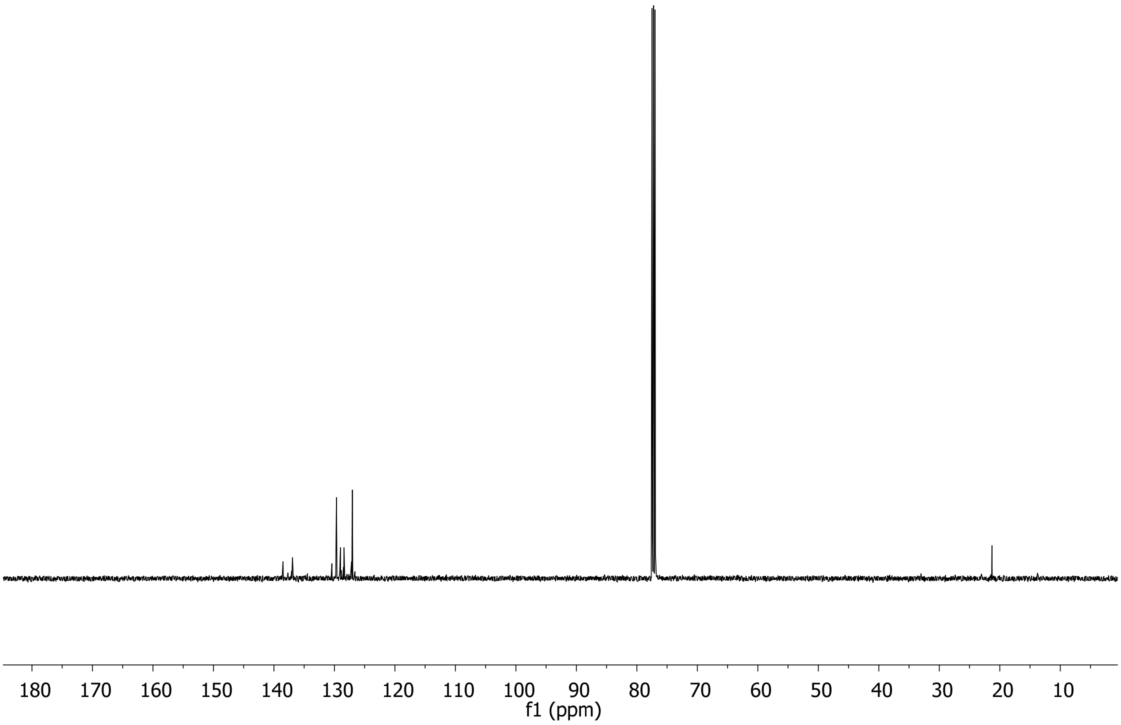

Figure S4. ^{13}C-NMR Spectra of the compound **3b,** in $CDCl_3$, 125 MHz

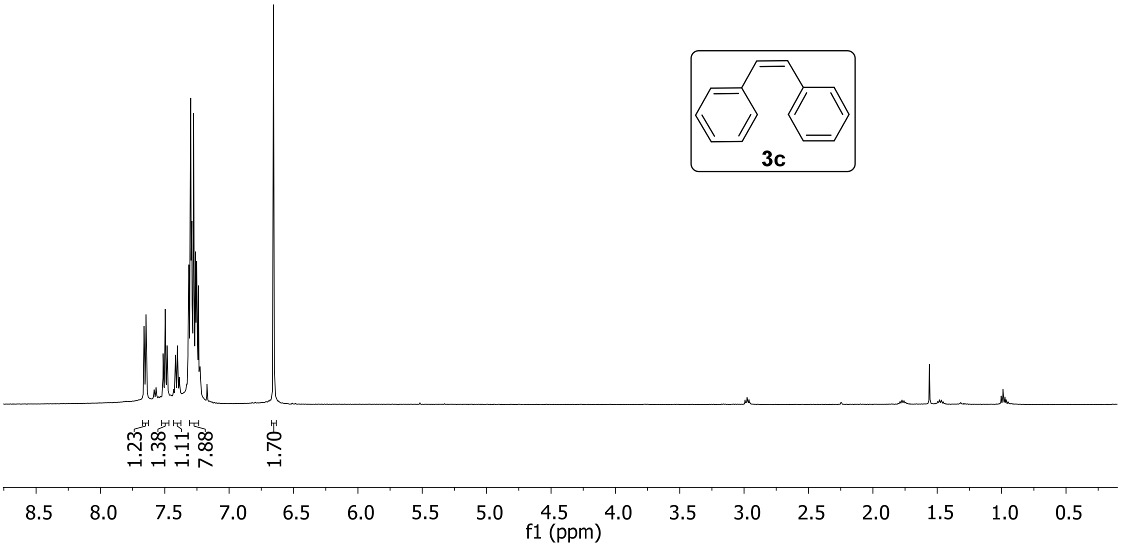

Figure S5. ^{1}H-NMR Spectra of the compound **3c,** in $CDCl_3$, 500 MHz

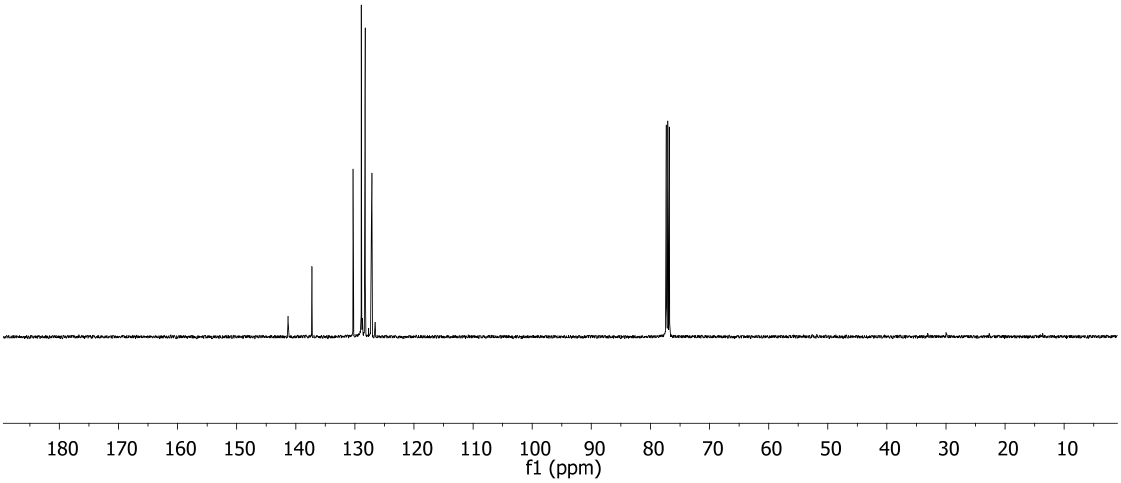

Figure S6. ^{13}C-NMR Spectra of the compound **3c,** in $CDCl_3$, 125 MHz

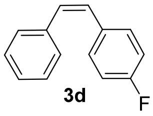

3d

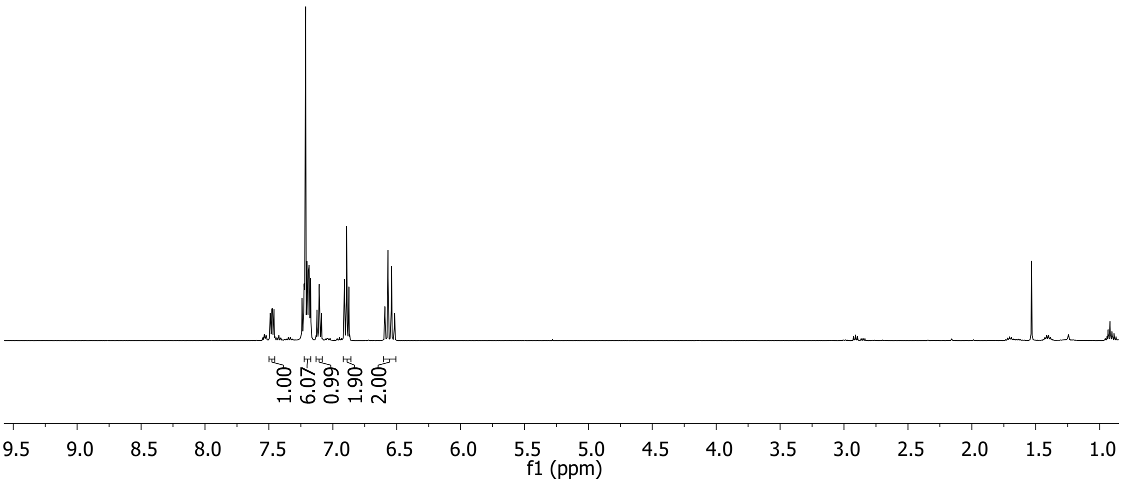

Figure S7. ^{1}H-NMR Spectra of the compound **3d,** in $CDCl_3$, 500 MHz

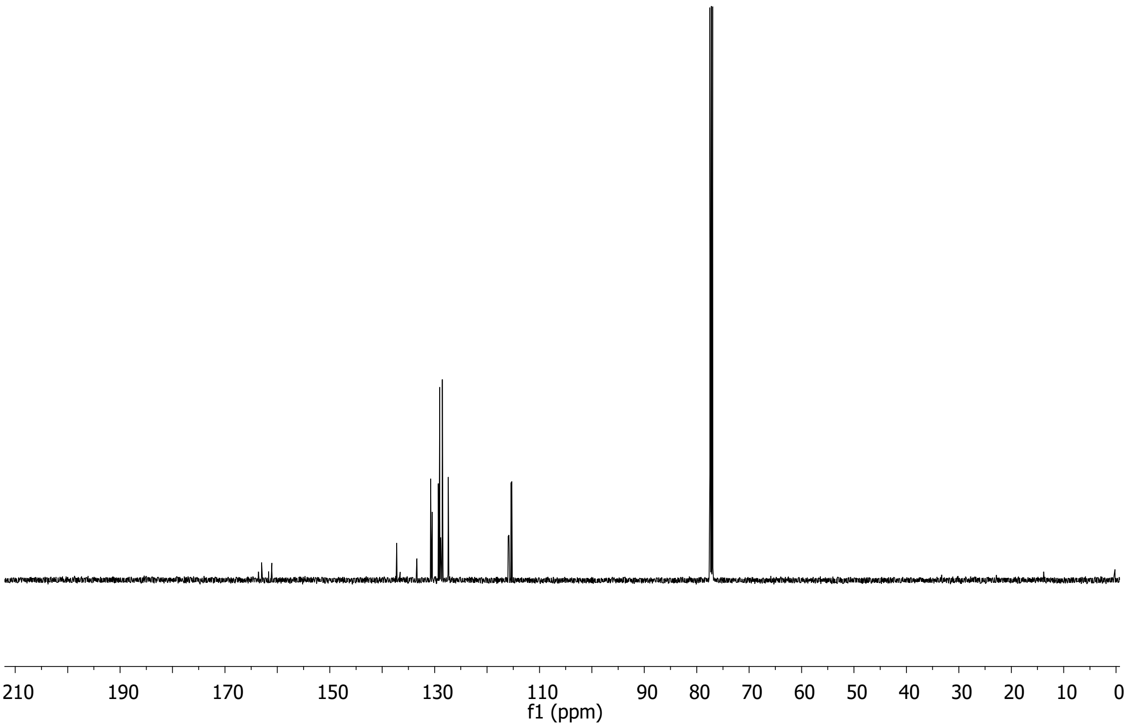

Figure S8. ^{13}C-NMR Spectra of the compound **3d,** in $CDCl_3$, 125 MHz

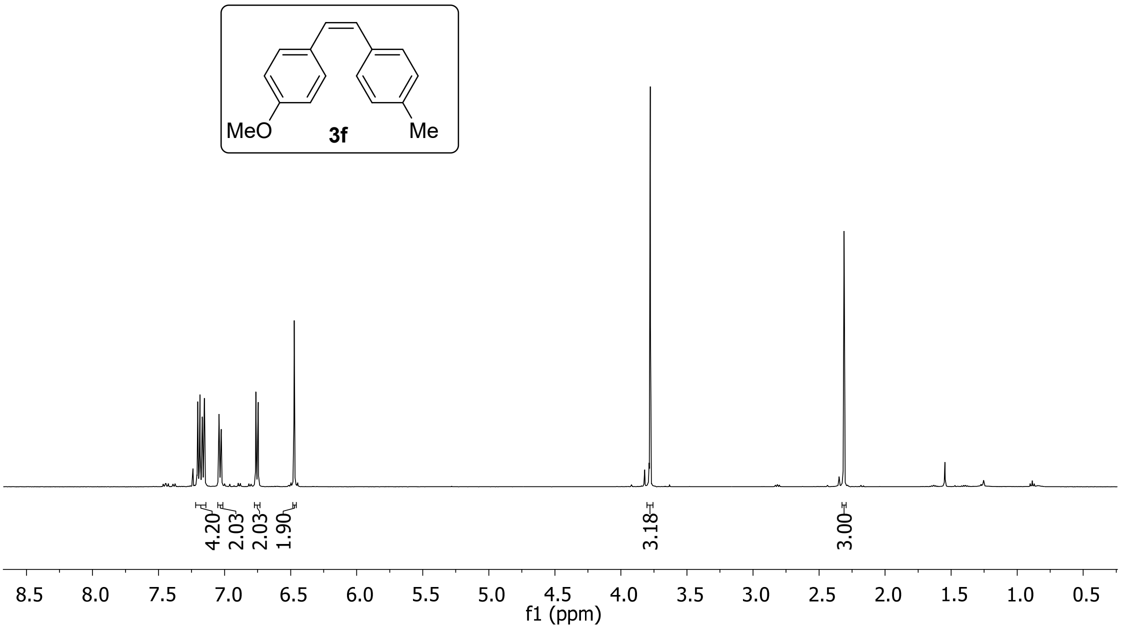

Figure S9. ¹H-NMR Spectra of the compound **3f,** in $CDCl_3$, 500 MHz

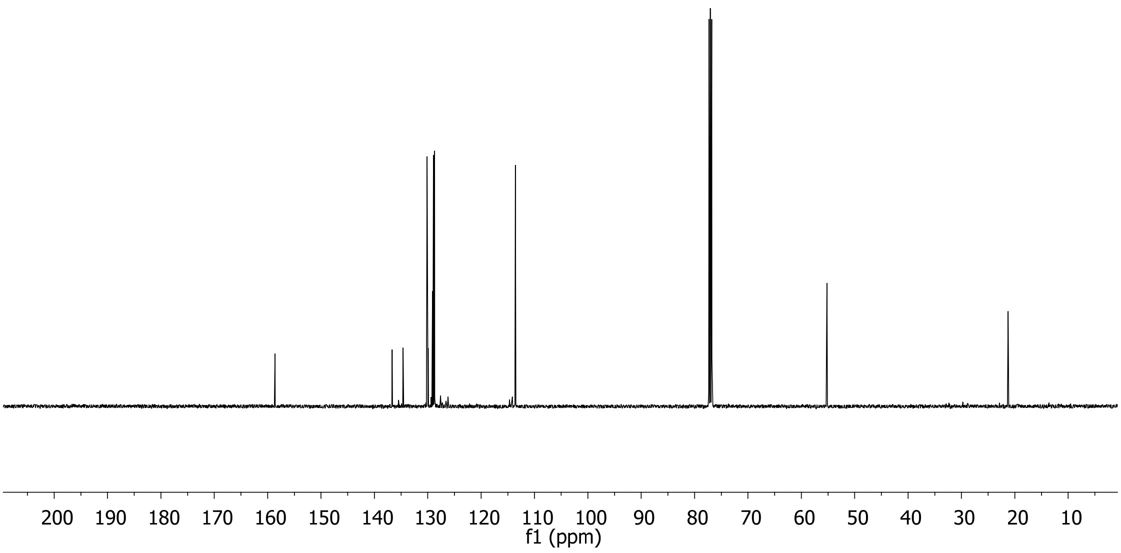

Figure S10. ¹³C-NMR Spectra of the compound **3f,** in $CDCl_3$, 125 MHz

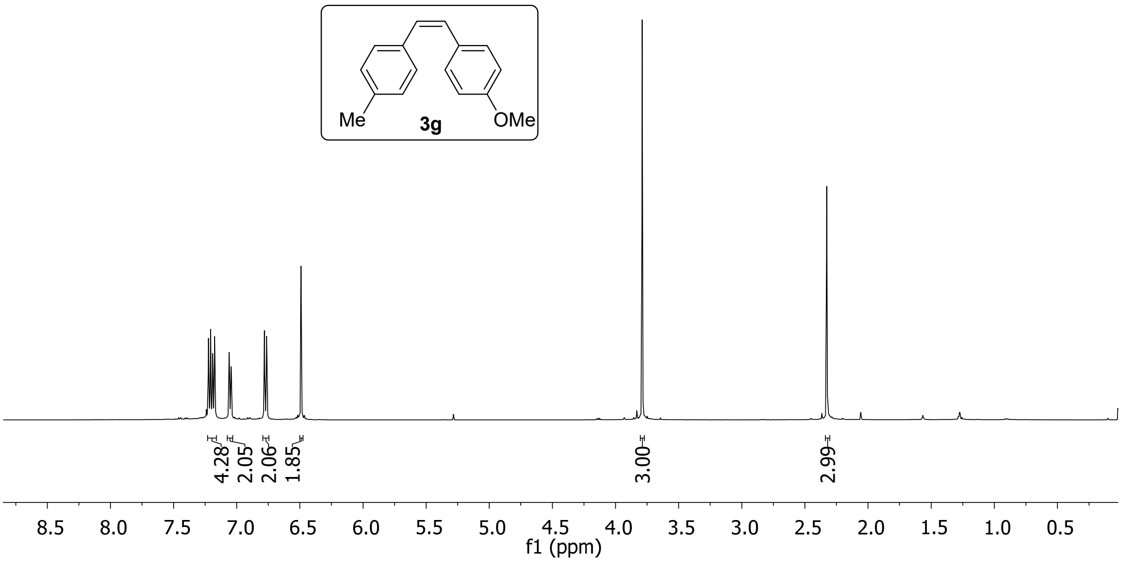

Figure S11. ^{1}H-NMR Spectra of the compound **3g,** in $CDCl_3$, 500 MHz

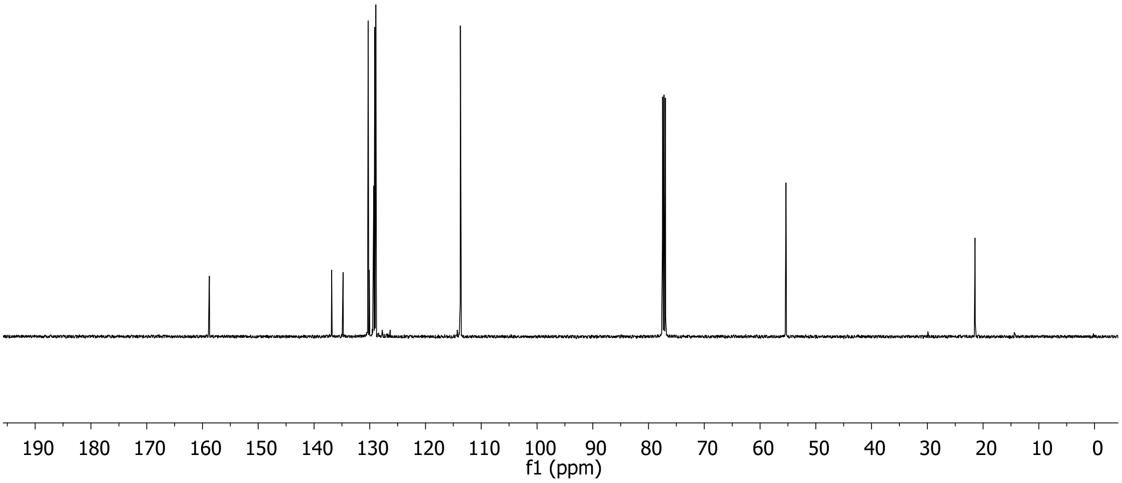

Figure S12. ^{13}C-NMR Spectra of the compound **3h,** in $CDCl_3$, 125 MHz

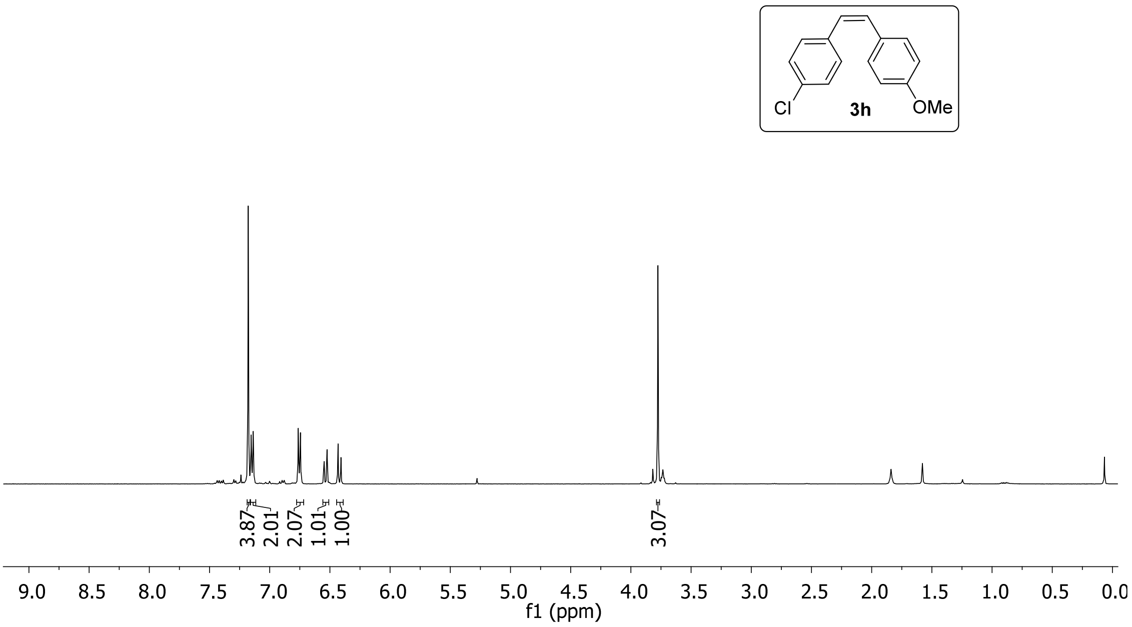

Figure S13. ^{1}H-NMR Spectra of the compound **3h,** in $CDCl_3$, 500 MHz

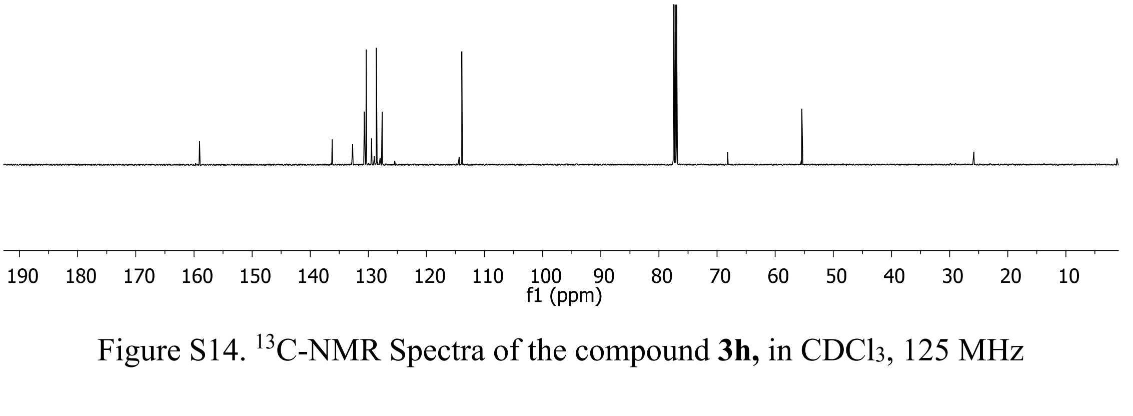

Figure S14. ^{13}C-NMR Spectra of the compound **3h,** in $CDCl_3$, 125 MHz

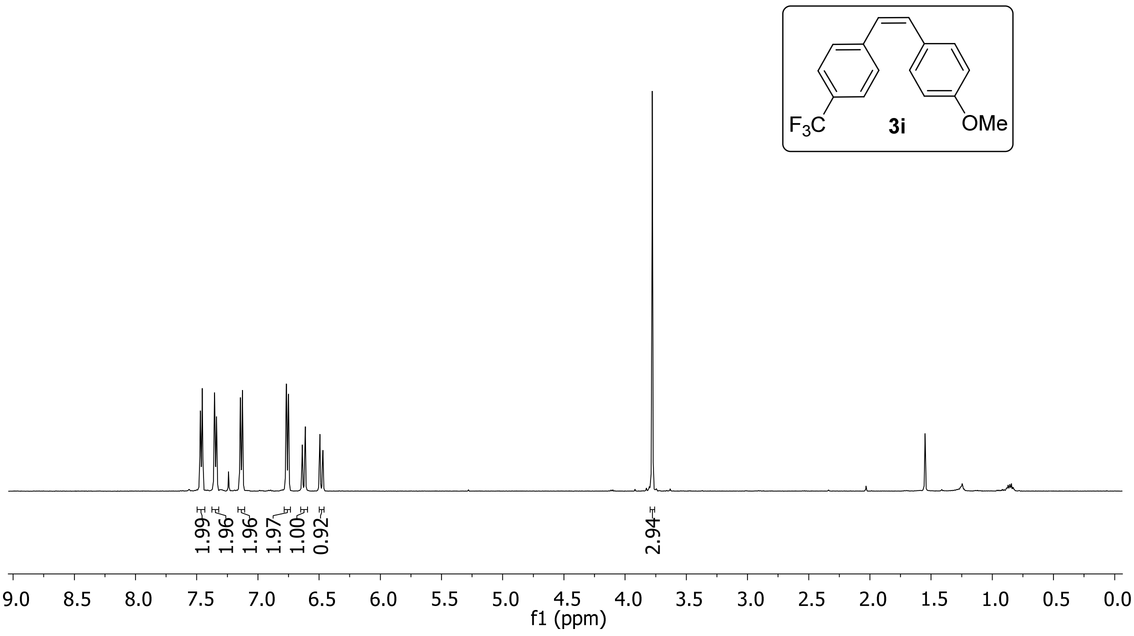

Figure S15. ^{1}H-NMR Spectra of the compound **3i,** in $CDCl_3$, 500 MHz

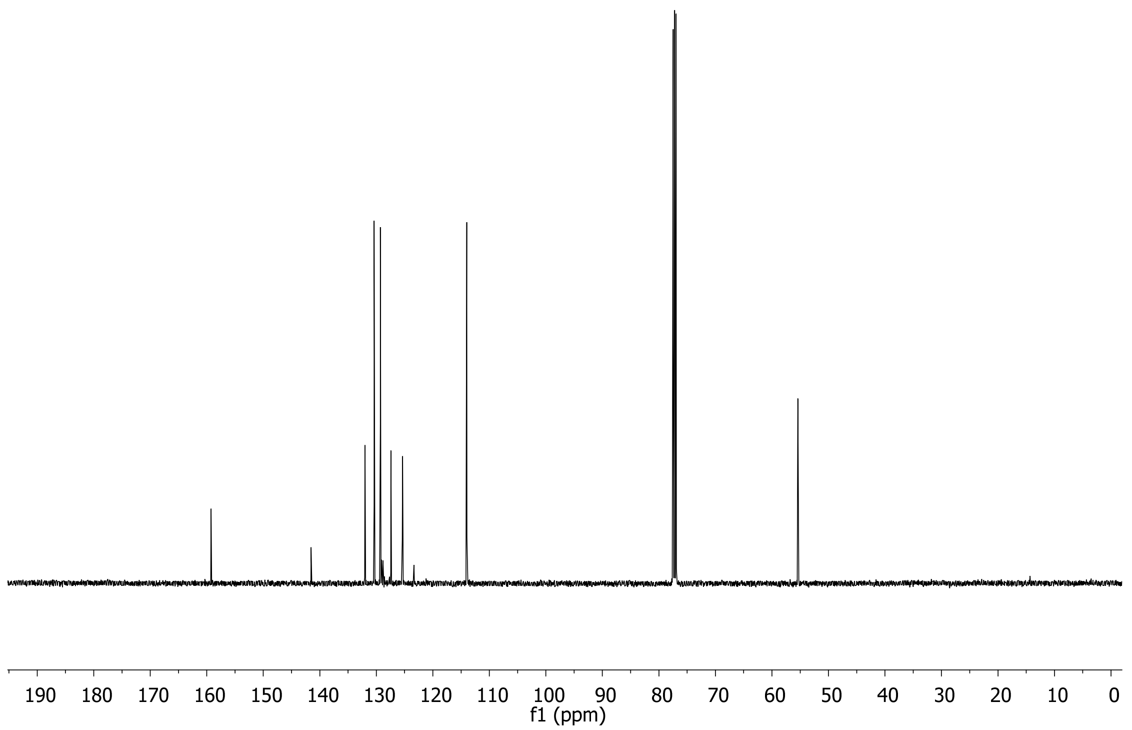

Figure S16. ^{13}C-NMR Spectra of the compound **3i,** in $CDCl_3$, 125 MHz

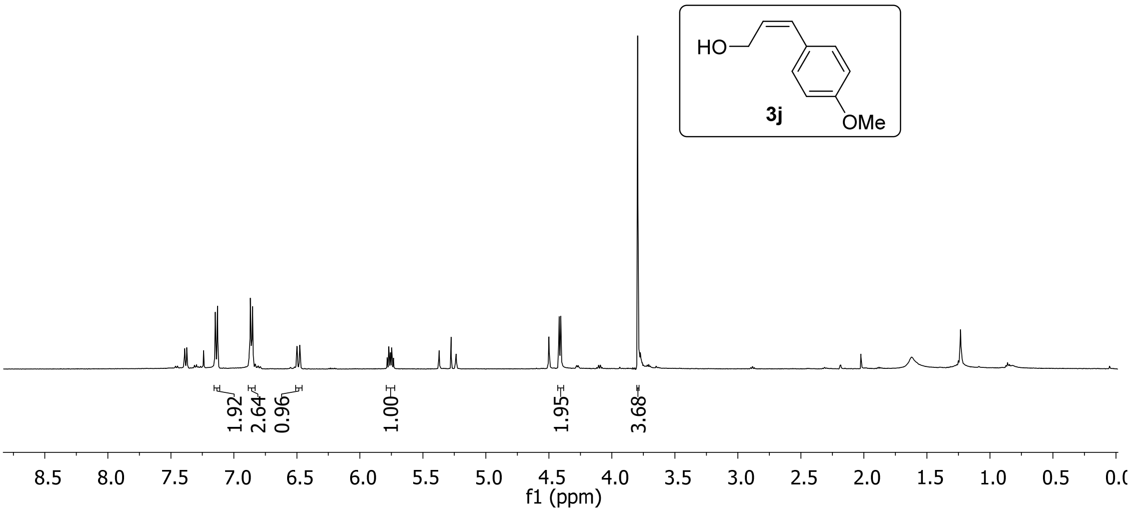

Figure S17. ^{1}H-NMR Spectra of the compound **3j,** in $CDCl_3$, 500 MHz

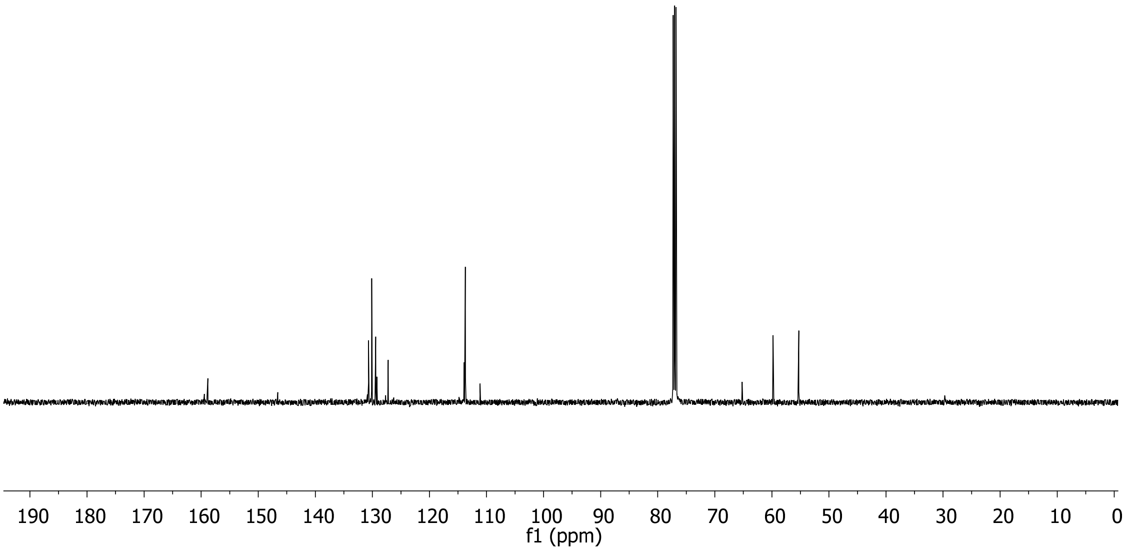

Figure S18. ^{13}C-NMR Spectra of the compound **3j,** in $CDCl_3$, 125 MHz

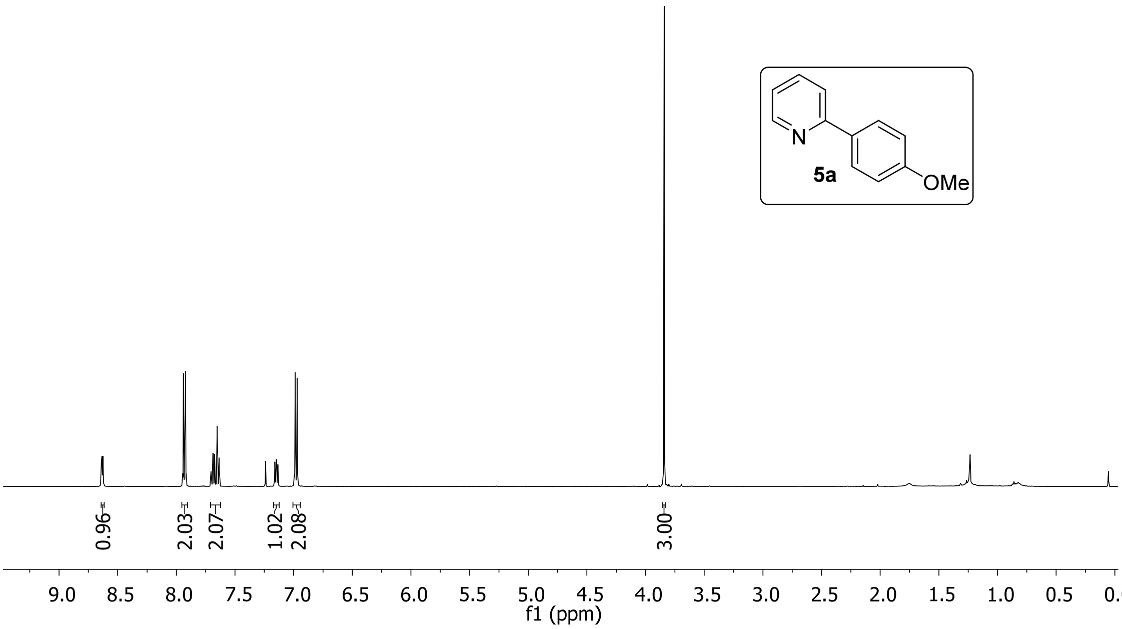

Figure S19. ^{1}H-NMR Spectra of the compound **5a,** in $CDCl_3$, 500 MHz

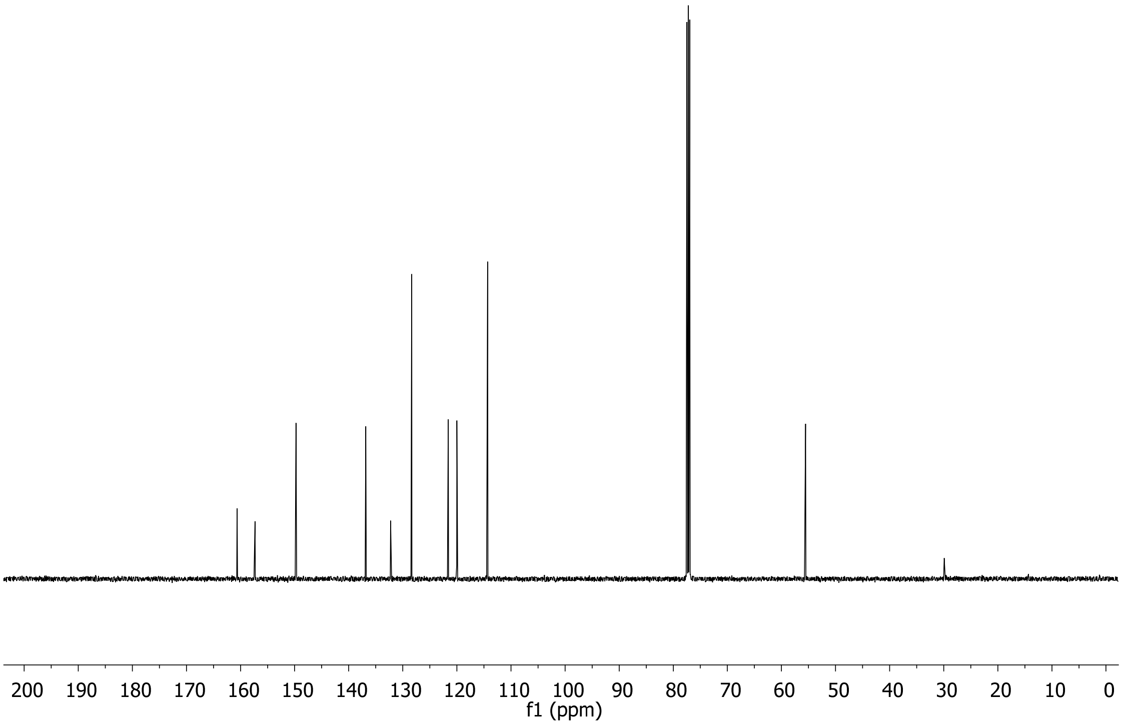

Figure S20. ^{13}C-NMR Spectra of the compound **5a,** in $CDCl_3$, 125 MHz

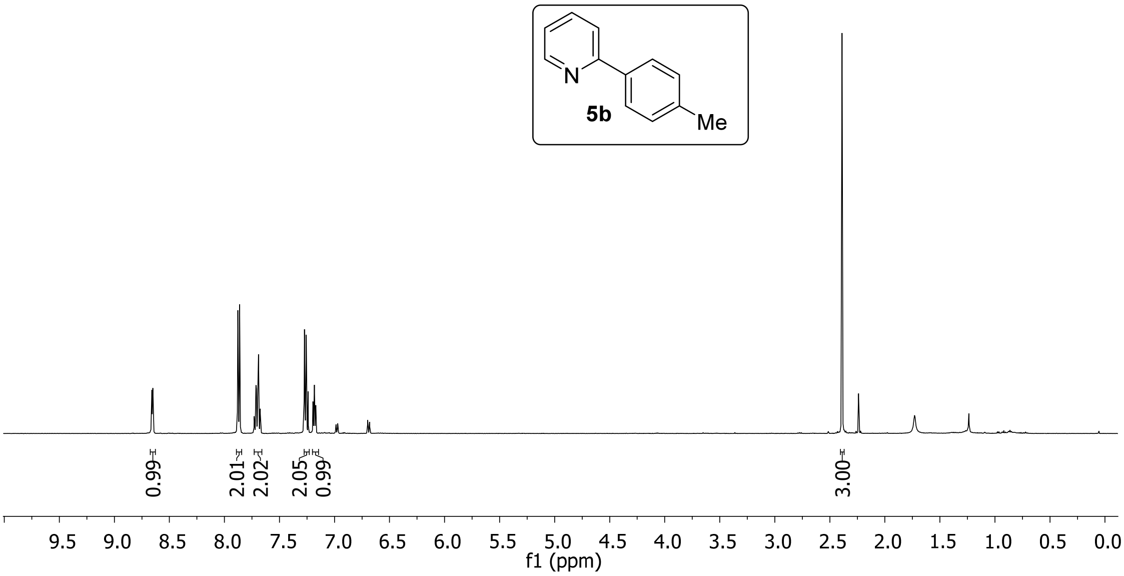

Figure S21. ^{1}H-NMR Spectra of the compound **5b,** in $CDCl_3$, 500 MHz

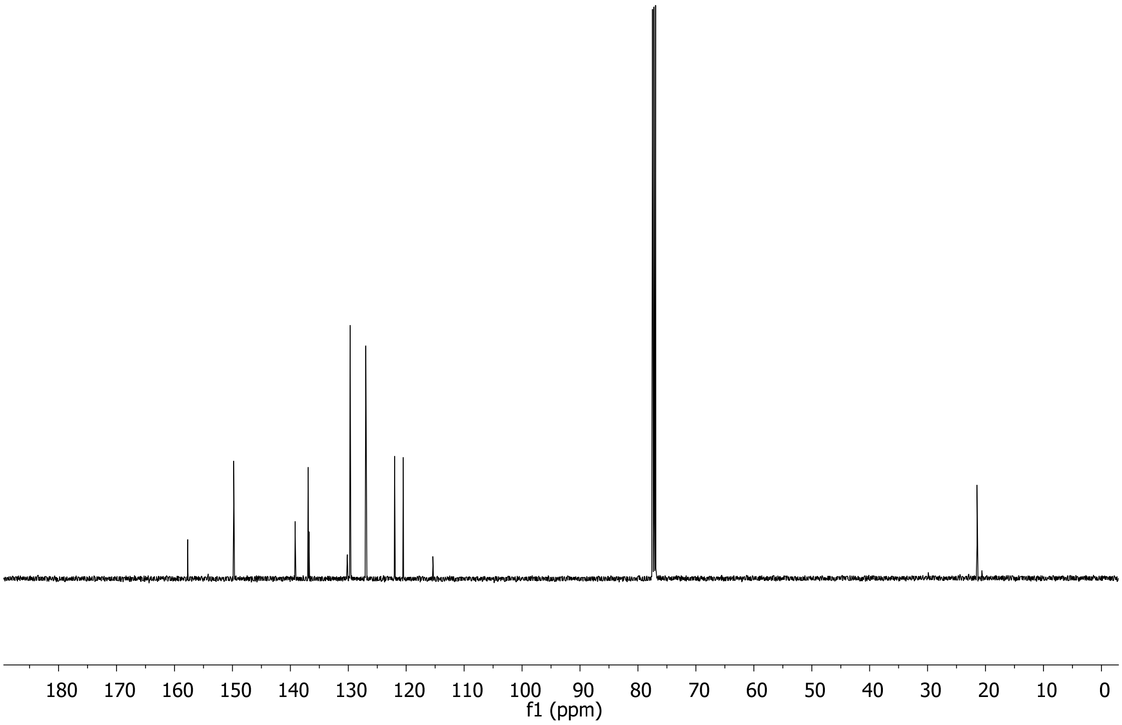

Figure S22. ^{13}C-NMR Spectra of the compound **5b,** in $CDCl_3$, 125 MHz

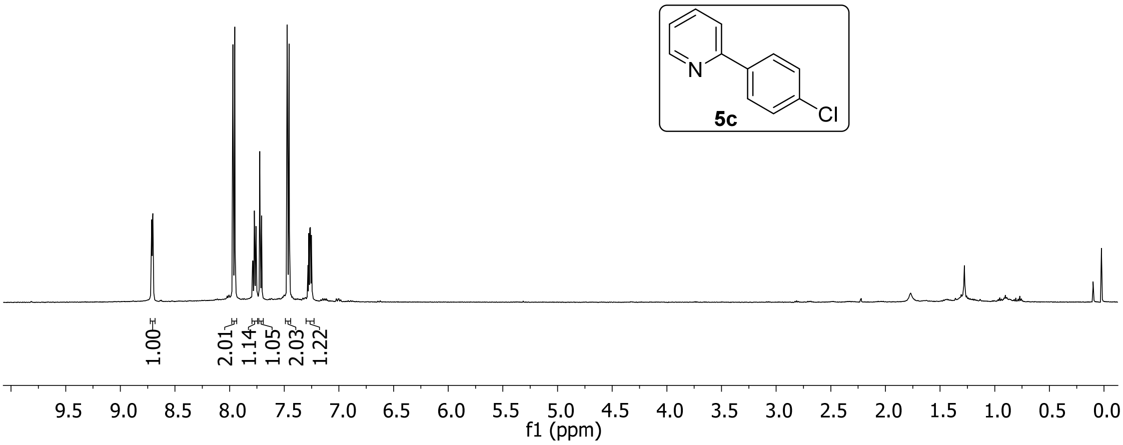

Figure S23. ^{1}H-NMR Spectra of the compound **5c,** in $CDCl_3$, 500 MHz

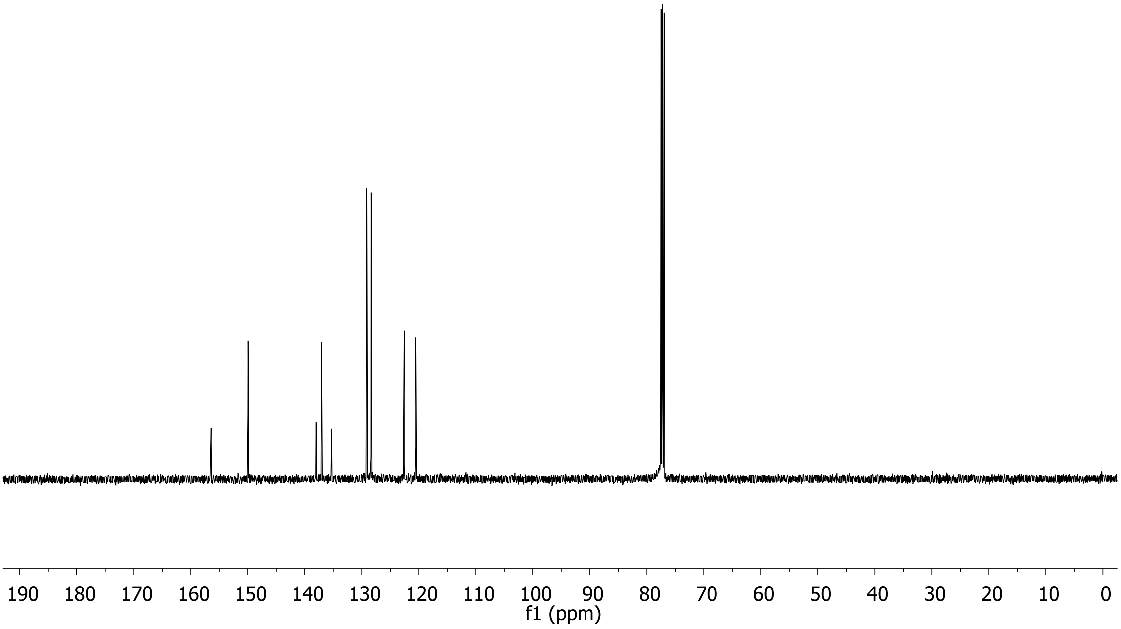

Figure S24. ^{13}C-NMR Spectra of the compound **5c,** in $CDCl_3$, 125 MHz

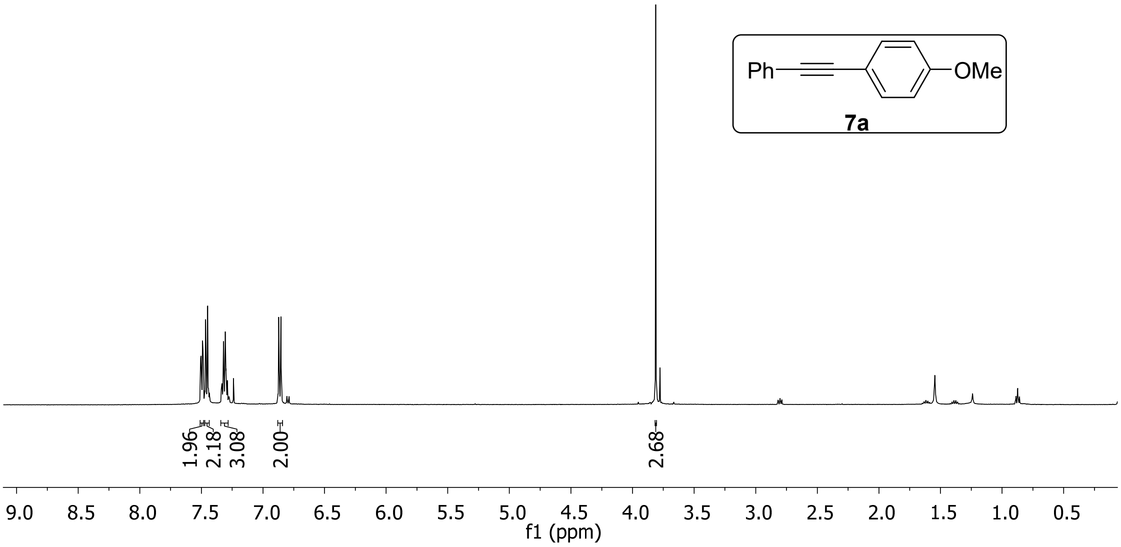

Figure S25. ^{1}H-NMR Spectra of the compound **7a,** in $CDCl_3$, 500 MHz

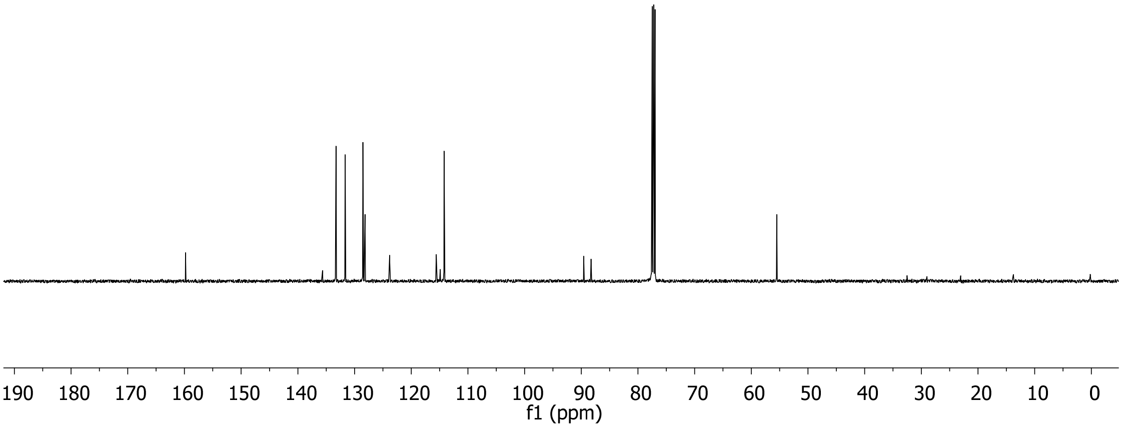

Figure S26. ^{13}C-NMR Spectra of the compound **7a,** in $CDCl_3$, 125 MHz

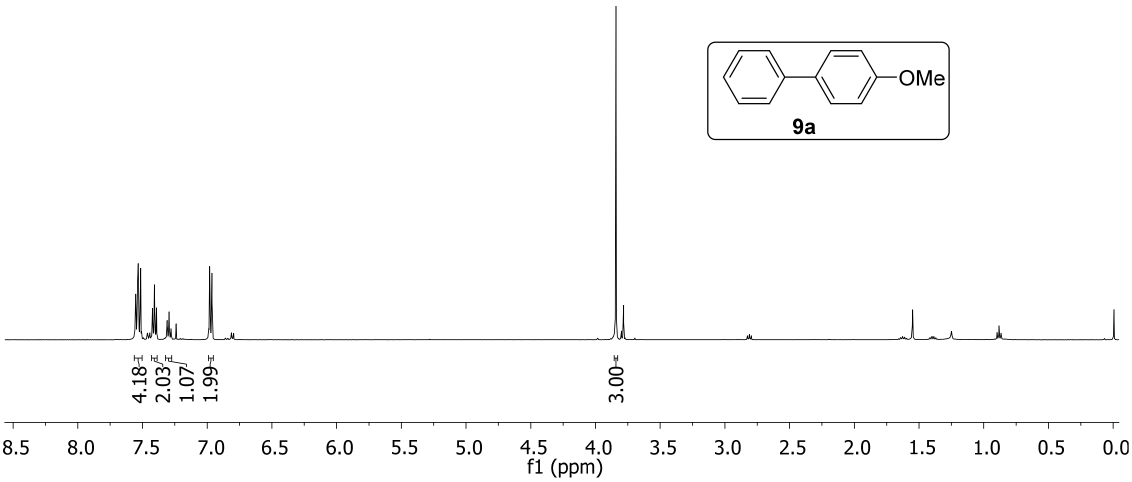

Figure S27. ^{1}H-NMR Spectra of the compound **9a,** in $CDCl_3$, 500 MHz

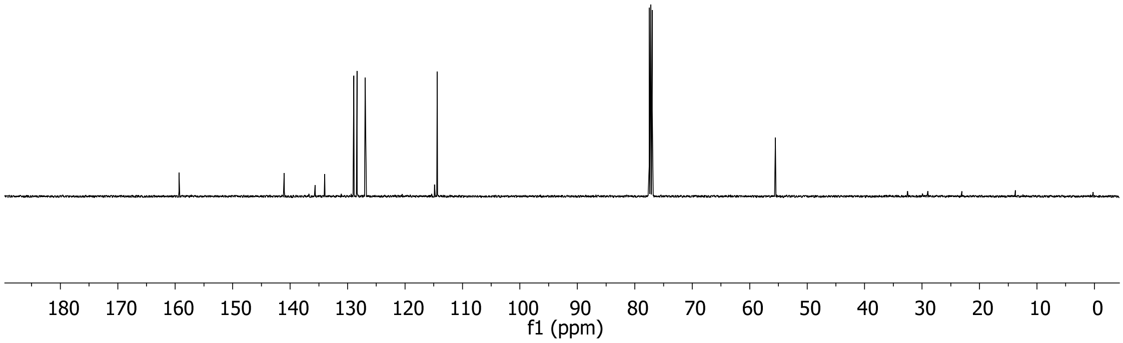

Figure S28. ^{13}C-NMR Spectra of the compound **9a,** in $CDCl_3$, 125 MHz
